# Sparsification of RNA structure prediction including pseudoknots

**DOI:** 10.1186/1748-7188-5-39

**Published:** 2010-12-31

**Authors:** Mathias Möhl, Raheleh Salari, Sebastian Will, Rolf Backofen, S Cenk Sahinalp

**Affiliations:** 1 Bioinformatics, Institute of Computer Science, Albert-Ludwigs-Universität, Freiburg, Germany; 2 Lab for Computational Biology, School of Computing Science, Simon Fraser University, Burnaby, BC, Canada; 3 Computation and Biology Lab, CSAIL, MIT, Cambridge MA, USA; 4 Centre for Biological Signalling Studies (bioss), Albert-Ludwigs-Universität, Freiburg, Germany

## Abstract

**Background:**

Although many RNA molecules contain pseudoknots, computational prediction of pseudoknotted RNA structure is still in its infancy due to high running time and space consumption implied by the dynamic programming formulations of the problem.

**Results:**

In this paper, we introduce sparsification to significantly speedup the dynamic programming approaches for pseudoknotted RNA structure prediction, which also lower the space requirements. Although sparsification has been applied to a number of RNA-related structure prediction problems in the past few years, we provide the first application of sparsification to pseudoknotted RNA structure prediction specifically and to handling gapped fragments more generally - which has a much more complex recursive structure than other problems to which sparsification has been applied. We analyse how to sparsify four pseudoknot structure prediction algorithms, among those the most general method available (the Rivas-Eddy algorithm) and the fastest one (Reeder-Giegerich algorithm). In all algorithms the number of "candidate" substructures to be considered is reduced.

**Conclusions:**

Our experimental results on the sparsified Reeder-Giegerich algorithm suggest a linear speedup over the unsparsified implementation.

## Background

Recently discovered catalytic and regulatory RNAs [1,2] exhibit their functionality due to specific secondary and tertiary structures [3,4]. The vast majority of computational analysis of non-coding RNAs have been restricted to nested secondary structures, neglecting pseudoknots - which are "among the most prevalent RNA structures" [5]. For example, Xaya-phoummine et al. [6] estimated that up to 30% of the base pairs in G+C-rich sequences form pseudoknots.

However the general problem of pseudoknotted RNA structure prediction is NP-hard. As a result, a number of approaches have been introduced for handling restricted classes of pseudoknots [7-13]. Condon *et al*. [14] give an overview of their structure classes and the algorithm-specific restrictions and Möhl *et al*. [15] develop a general framework showing that all these algorithms follow a general scheme, which they use for efficient alignment of pseudoknotted RNA.

The most general algorithm (with respect to the pseudoknot classes handled) among the above by Rivas and Eddy (R&E) has a running time of *O*(*n*^6^) time and space consumption of *O*(*n*^4^). It is therefore too expensive to directly apply this algorithm for large scale data analysis. Unfortunately, even the most efficient algorithm by Reeder and Giegerich (R&G) still has a high running time of *O*(*n*^4^), although it strongly restricts the class of predictable pseudoknots.

In this paper we introduce the technique of sparsification to the problem of pseudoknotted RNA structure prediction. Sparsification improves the expected running time and space usage of a dynamic programming based structure prediction algorithm without introducing additional restrictions on the structure class handled or compromising the optimality of solutions. Sparsification has been recently applied to improve time and space complexity of various existing RNA-related structure prediction algorithms. In particular, it turned out to be successful for RNA folding for pseudoknot-free structures [16,17], simultaneous alignment and folding [18] as well as RNA-RNA interaction prediction [19].

### Contributions

We study sparsification of pseudoknotted RNA structure prediction. Algorithms developed for this problem differ from the previously sparsified algorithms by their use of gapped fragments and their more complex recursion structure. Our main contribution in this paper is the solution to the algorithmic challenges due to this increased complexity. Among all DP based pseudoknot prediction algorithms, we focus on the fastest algorithm (R&G) and the most general one (R&E) and develop sparse variants of these dynamic programming algorithms. Furthermore, we consider sparsification of the algorithm by Akutsu *et al*. and Uemura *et al*. (A&U) [9,10] as well as the algorithm by Dirks and Pierce (D&P) [12]. Due to sparsification, the resulting algorithms need to consider only a limited number of candidates substructures compared to the original algorithms. As a result, we analyze the theoretical worst case complexities in terms of the number of candidate substructures. We also present experimental results, comparing our implementations of the original and sparsified *R*&*G *algorithm. These results suggest a significant (roughly a linear factor) reduction in the number of candidates over the original algorithm.

## Methods

### Sparsification of the Reeder and Giegerich algorithm

The R&G algorithm [13] predicts the minimum free energy structure allowing canonical pseudoknots for a sequence *S *of length *n*. It extends the Zuker algorithm by adding one more matrix *K *(for knot), where *K*(*i*, *j*) denotes the energy for the best *canonical *pseudoknot that starts at position *i *and ends at position *j*. Note that the original presentation of the algorithm in terms of the ADP framework does not explicitly consider a matrix K but only a motif *knot*. Canonical pseudoknots are defined as follows. Each pair of base pairs *p*_1 _= (*i*, *i'*) and *p*_2 _= (*j'*, *j*) with *i *<*j' *<*i' *<*j *induces one canonical pseudoknot that consists of two crossing stems {(*i*, *i'*), (*i*+1, *i'- *1),..., (*i*+*d*_*i*, *i' *_- 1, *i'*- *d*_*i*, *i' *_+1)} and {(*j'*, *j*), (*j' *+ 1, *j *- 1),..., (*j' *+ *d*_*j'*, *j *_- 1, *j *- *d*_*j'*, *j *_+ 1)} where the stacking length of the two stems, *d*_*i*, *i' *_and *d*_*j'*, *j*_, respectively, is maximally extended as long as all base pairs are valid Watson-Crick base pairs.

To allow for sparsification, we restrict the scoring scheme slightly such that the energy of a canonical pseudoknot only depends on the left ends of its base pairs and hence can be described as *PK-Energy*(*i*, *d*_*i*, *i'*_, *j'*, *d*_*j'*, *j*_). This implies that the scoring scheme does not distinguish between G-C and G-U base pairs in pseudoknot-stems, since their left ends are identical. Then,

(1) $$K(i,j)=\underset{i^{'},j^{'}}{\min}score(i,j^{'},i^{'},j)$$

with

(2) $$\begin{array}{l} score(i,j^{'},i^{'},j)=\\ \;PK­Energy(i,d_{i,i^{'}},j^{'},d_{j^{'},j})+W(i+d_{i,i^{'}},j^{'}-1)+\\ \;W(j^{'}+d_{j^{'},j},i^{'}-d_{i,i^{'}})+W(i^{'}+1,j-d_{j^{'},j}). \end{array}$$

**Figure 1:**
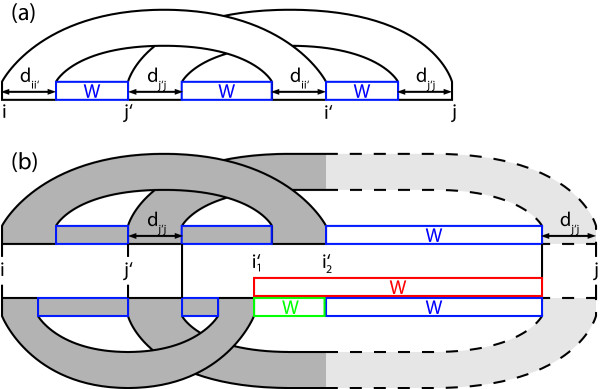
**Recursion for canonical pseudoknots (a) and their sparsification (b)**.

As shown in Figure 1(a), for each canonical pseudoknot starting at *i *and ending at *j *the recursion decomposes into the pseudoknot itself and the three fragments in-between its two crossing stems. Such pseudoknots add one case in the computation of a matrix entry *W*(*i*, *j*), which, as in the Zuker algorithm, contains the optimal energy of a substructure starting at position *i *and ending at position *j*. Due to the restriction to canonical pseudoknots, the recursion of R&G minimizes only over all possible instances of *i' *and *j'*, because the maximal stacking lengths *d*_*i*, *i' *_and *d*_*j'*, *j *_are uniquely determined once *i' *and *j' *are fixed. Furthermore, Reeder and Giegerich note that the maximal stacking length *d*_*x*, *y *_can be precomputed for all *x*, *y *in *O*(*n*^3^) time and stored in an *O*(*n*^2^) table.

In order to sparsify the algorithm, we develop an appropriate notion of a *candidate *such that it is not necessary to minimize over all possible *i' *and *j' *but only over the candidates.

#### Definition 1 (R&G candidate)

*Let *$i<j^{'}<{i^{'}}_{1}<{i^{'}}_{2\;}$*and *$d_{j^{'},j}\leq {i^{'}}_{1^{'}}-j^{'}$. *Then *${i^{'}}_{1}$*dominates *${i^{'}}_{2}$*with respect to *(*i*, *j' d*_*j'*, *j*_), *iff*

$${\text{score}}_{{i^{'}}_{2}}(i,j^{'},{i^{'}}_{2})\geq {\text{score}}_{{i^{'}}_{2}}(i,j^{'},{i^{'}}_{1}),$$

where

$$\begin{array}{l} {\text{score}}_{i_{c}}(i,j^{'},i^{'}):=\\ \;PK­Energy(i,d_{i,i^{'}},j^{'},d_{j^{'},j})+W(i+d_{i,i^{'}},j^{'}-1)\\ \;+W(j^{'}+d_{j^{'},j},i^{'}-d_{i,i^{'}})+W(i^{'}+1,{i^{'}}_{c}). \end{array}$$

*We say that *${i^{'}}_{2}$*is a candidate with respect to *(*i*, *j'*, *d*_*j'*, *j*_) *if there does not exist any *${i^{'}}_{1}$*that dominates it*.

The notion of a candidate is visualized in Figure 1(b). There, ${i^{'}}_{1}$ dominates ${i^{'}}_{2}$ if the score for the gray area at the top (including the dashed part whose exact position is not determined) is not better than the score for the corresponding gray area at the bottom plus the green part. Note that these scores (and hence the candidate *i'*) depend only on *i*, *j'*, and *d_j',j _*and are independent of *d_i,i' _*and *j*. The following lemma shows that the notion of a candidate given in Def. 1 is suitable for sparsification, i.e. some *i' *needs to be considered in the recursion (for all *j*) only if it is a candidate, because otherwise it is dominated by a candidate that yields a better score.

#### Lemma 1 (R&G sparsification)

*Let *${i^{'}}_{2}$*be dominated by *${i^{'}}_{1}$*with respect to some *(*i*, *j'*, *d*_*j'*, *j*_). *Then for all j it holds *$score(i,j^{'},{i^{'}}_{1},j)\leq score(i,j^{'},{i^{'}}_{2},j)$.

Proof We start with the inequality of Def. 1 and add $W({i^{'}}_{2}+1,j-d_{j^{'},j})$ on both sides.^. ^Then the claim follows immediately from $W({i^{'}}_{1}+1,j-d_{j^{'},j})\leq W({i^{'}}_{1}+1,{i^{'}}_{2})+W({i^{'}}_{2}+1,j-d_{j^{'},j})$. In Figure 1(b) this corresponds to the fact that the score for the red box is at least as good as the score from the green and the blue box together. This triangle inequality holds by the correctness of the (unsparsified) algorithm: For all *x < y < z *we have *W*(*x*, *y*)+*W*(*y*+1, *z*) ≤ *W*(*x*, *z*) since the concatenation of the best structures for the ranges (*x*, *y*) and (*y*, *z*) always forms a valid structure for the range (*x*, *z*) with score *W*(*x*, *y*)+*W*(*y*+1, *z*) which is hence never better than the optimal score *W*(*x*, *z*) for that range.   □

The sparsified algorithm maintains lists *L_i _*of candidates for each pair (*j'*, *d*_*j'*, *j*_) since only the lists for one *i *need to be maintained in memory at the same time. Whenever in the computation of some *score*(*i*, *j'*, *i'*, *j*) the *i' *is considered the first time for this *i *and *j'*, it is checked whether it is a candidate and if so, it is added to the respective list. For all other instances of *j*, *i' *is then considered only if it is contained in the list. The sparsified algorithm is given by the following pseudo-code (*n *:= |*S|*).

1: **for ***i *:= *n *to 1 **do**

2:   **for all ***d*_*j'*, *j*_, *j' *≤ *n ***do**

3:      *L_i_*(*j'*, *d*_*j'*, *j*_) := empty list;

4:   **end for**

5:   **for ***j *:= *i *+ 3 to *n ***do**

6:      *K*(*i*, *j*) := ∞

7:      **for ***j' *:= *i *+ 1 to *j - *2 **do**

8:         *// check new elements for candidacy*

9:         **for **$i_{c}:=\max\{j^{'}+d_{j^{'},j},\;\;{\text{checked}}_{i,j^{'},d_{j^{'},j}}+1\}$ to *j *- *d*_*j'j *_**do**

10:            **if **${\text{score}}_{i_{c}}(i,j^{'},i_{c})<{\text{score}}_{i_{c}}(i,j^{'},i^{'})$ for all *i' *∈*L_i_*(*j'*, *d*_*j'*, *j*_) **then**

11:               add *i_c _*to *L_i_*(*j'*, *d*_*j'*, *j*_)

12:            **end if**

13:         **end for**

14:      ${\text{checked}}_{i,j^{'},d_{j^{'},j}}:=\max({\text{checked}}_{i,j^{'},}{}_{d_{j^{'},j}},j-d_{j^{'},j})$

15:      *// iterate over all candidates*

16:      *K*_*i*, *j'*, *j *_:= ∞

17:      **for all ***i' *∈ *L_i_*(*j'*, *d*_*j'*, *j*_) **do**

18:         *K*_*i*, *j'*, *j *_:= min {*K*_*i*, *j'*, *j*_, *score*(*i*, *j'*, *i'*, *j*)}

19:      **end for**

20:      *K*(*i*, *j*) := min {*K*(*i*, *j*), *K*_*i*, *j'*, *j*_}

21:   **end for**

22:   compute matrix entries *V *(*i*, *j*) and *W*(*i*, *j*) as in Wexler *et al*.

23:      *W*(*i*, *j*) := min(*W*(*i*, *j*), *K*(*i*, *j*))

24:   **end for**

25: **end for**

The candidate lists are initialized in line 2. In lines 7 to 11 all new values *i_c _*that have not been considered so far, are tested for candidacy. Here, ${\text{checked}}_{i,j^{'},d_{j^{'},j}}$ denotes the largest *i' *that has been checked for candidacy in list *L_i_*(*j'*, *d*_*j'*, *j*_).

Lines 14 to 17 compute scores *score*(*i*, *j'*, *i'*, *j*) for all candidates *i'*. In line 20, we compute *W*(*i*, *j*) and *V*(*i*, *j*) as in the sparsified pseudoknot-free structure prediction approach due to Wexler *et al*. [16]. The computation of matrices *K *and *W *is interleaved such that all entries *K*(*i*, *j*) and *W*(*i*, *j*) are computed before all entries *K*(*i'*, *j'*) and *W*(*i'*, *j'*) for *i ≤ i' ≤ j' ≤ j *and *i *≠ *i' *or *j *≠ *j'*.

#### Complexity Analysis

Whereas the original algorithm requires *O*(*n*^4^) time (for *n *= |*S*|), the sparsified variant requires *O*(*n*^3^*L*) time where *L *is the total size for all candidate lists of some *i *i.e. $L:=\max_{i}\sum_{j',d_{j'}{{}_{,}}_{j}}\left|L_{i}(j',d_{j'.j})\right|$. Obviously, *L ≤ n*. In order to maintain the asymptotic space complexity *O*(*n*^2^) of the original algorithm, we do not maintain all lists *L_i_*(*j'*, *d*_*j'*, *j*_) in memory but only the lists with *d*_*j'*, *j *_≤ *k *where *k *> 0 is a small constant. Please note that to keep presentation simple, we didn't make this explicit in the pseudo-code. Since the maximal stacking length is usually small, there are only very few instances of *j *with *d*_*j'*, *j *_>*k *such that for those few *j *it is cheap to consider all *i' *as candidates. Hence, we store *O*(*kn*) = *O*(*n*) candidate lists each requiring at most *O*(*n*) space.

Wexler *et al*. [16] use the assumption that RNA folding satisfies the polymer-zeta property to derive a tighter bound on the expected-case asymptotic complexity. However, we focus on the practical speed-up that is obtained by our implementation due to the following reasons. First, it is unclear whether the energy-models for pseudoknot prediction exhibit this property and second it is unclear whether the asymptotic behaviour already appears in the feasible range of input sizes. As shown in the results, the sparsified variant runs two to four times faster than the unsparsified variant for input sizes up to 1000 nucleotides.

### Sparsification of the Rivas and Eddy Algorithm

The class of structures predicted by the R&E algorithm [8], here called class of R&E structures, is the most general RNA secondary structure prediction algorithm described in the literature [14]. To keep presentation simple we explain the sparsification strategy for a base-pair maximization algorithm that handles the R&E structure class. Finally, we motivate that sparsification can be transferred to the R&E energy minimization algorithm.

First, we give recursions of base pair maximization for R&E structures. Note that the recursions are intentionally very close to the recursions of the R&E energy minimization algorithm. After initialization for *i *≥ *j *and *k *≥ *l*

$$W(i,j)=\{\begin{array}{ll} 0 & \text{if}\;i=j\;\text{or}\;i=j+1\\ -\infty & \text{if}\;i>j+1 \end{array}$$

and

W(i,j;k,l)=−∞ if j<i or l<kW(i,i;k,k)=bp(i,k)

Where $\text{bp}(i,j)=\{\begin{array}{ll} 1 & \text{if}\;S_{i},S_{k}\;\text{complementary}\\ -\infty & \text{otherwise}, \end{array}$ is the *base pair contribution*, the recursions (R&E recursions) are given for 1 ≤ *i *<*j *<*k *<*l *≤ |*S*| as

$$\begin{array}{l} W(i,j)=\max\\ \{\begin{array}{ll} W(i,j-1) & \left(12'\right)\\ \text{bp}(i,j)+W(i+1,j-1) & \left(1'21\;'\right)\\ \max_{j^{'}}W(i,j^{'}-1)+W(j^{'},j) & \left(12\right)\\ \max_{j^{'},k^{'},l^{'}}\left(\begin{array}{l} W(i,j^{'}-1;k^{'}+1,l^{'}-1)\\ +W(j^{'},k^{'};l^{'},j) \end{array}\right) & \left(1212\right) \end{array} \end{array}$$

$$\begin{array}{l} W(i,j;k,l)=\max\\ \{\begin{array}{ll} W(i+1,j;k,l) & \left(1'2\text{G}2\right)\\ W(i,j-1;k,l) & \left(12'\text{G1}\right)\\ W(i,j;k+1,l) & \left(1\text{G}2'1\right)\\ W(i,j;k,l-1) & \left(\text{1G}12'\right)\\ \max_{j^{'}}W(i,j^{'})+W(j^{'}+1,j;k,l) & \left(12\text{G}2\right)\\ \max_{j^{'}}W(i,j^{'}-1,j;k,l)+W(j^{'},j) & \left(12\text{G}1\right)\\ \max_{l^{'}}W(i,j;l^{'}+1,l)+W(k,l^{'}) & \left(\text{1G}21\right)\\ \max_{l^{'}}W(i,j;k,l^{'}-1)+W(l^{'},l) & \left(\text{1G}12\right)\\ \max_{j',k'}\left(\begin{array}{l} W(i,j^{'}-1;k^{'}+1,l)\\ +W(j^{'},j;k,k^{'}) \end{array}\right) & \left(12\text{G}21\right)\\ \max_{j',k^{'}}\left(\begin{array}{l} W(i,j^{'}-1;k,k^{'}-1)\\ +W\left(j^{'},j;k^{'},l\right) \end{array}\right) & \left(12\text{G12}\right)\\ \max_{k^{'},l^{'}}\left(\begin{array}{l} W\left(i,j;k^{'}+1,l^{'}-1\right)\\ +W\left(k,k^{'};l^{'},l\right) \end{array}\right) & \left(1\text{G}2\text{12}\right)\\ \max_{i^{'},j^{'}}\left(\begin{array}{l} W(i,i^{'}-1;j^{'}+1,j)\\ +W\left(i^{'},j^{'};k,l\right) \end{array}\right) & \left(121\text{G}2\right). \end{array} \end{array}$$

**Figure 2:**
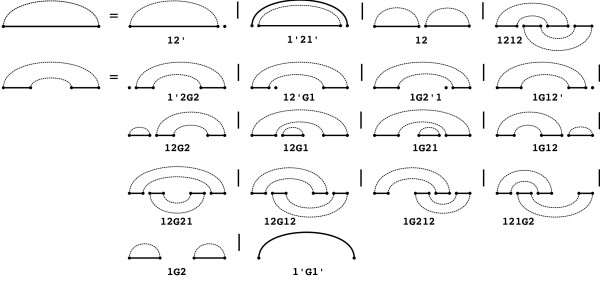
**Decomposition for R&E base pair maximization annotated with labels, i.e. split types, of the corresponding recursion cases**.

It is easy to check that *W*(1, |*S*|) is the maximal number of base pairs in a *R*&*E *structure of *S*, because the recursions perform the same decompositions as the original R&E recursions. Note that *W*(*i*, *j*; *k*, *l*) is the maximal number of base pairs in structures with at least one base pair that spans the gap. We label each recursion case in a way that illustrates the type of the decomposition of this case. The idea of these labels is taken from Möhl *et al*. [15], where we developed a type system for decompositions, which there are called splits. For this reason, we call these labels split types, however, we won't need any details of the typing system. The decomposition by R&E is illustrated in Figure 2.

A *fragment *is defined as a set of positions of the fixed sequence *S*. The fragments corresponding to matrix entries in the *R*&*E *recursion can be described conveniently by their boundaries. We distinguish *ungapped fragments F *= {*i*,...,*j*}, written (*i*, *j*), and *1-gap fragments F' *= {*i*,...,*j*} ∪ {*k*,...,*l*}, written (*i*, *j*; *k*, *l*) where *i*, *j*, *k*, *l*, are called *boundaries *of respective *F *or *F'*. A *split *of a fragment *F *is a tuple (*F*_1_, *F*_2_) such that *F *= *F*_1 _∪ *F*_2 _and *F*_1 _∩ *F*_2_∅.

For our sparsification approach, we will show that in each recursion case, certain optimally decomposable fragments do not have to be considered for computing an optimal solution, because each decomposition using these fragments can be replaced by a decomposition using a smaller fragment. We define optimal decomposability with respect to the split type of a R&E recursion case.

#### Definition 2 (Optimally decomposable)

*A fragment F is *optimally decomposable by a split of type *T *(*T*-OD) *iff there is a split *(*F*_1_, *F*_2_) *that occurs in recursion case T and W*(*F*_1_) + *W *(*F*_2_) ≥ *W *(*F *).

*A fragment F is *optimally decomposable w.r.t a set of split types T(T-OD)*iff F is T-OD for some *T∈T.

Here, we emphasize that testing *T*-OD for a fragment *F *is simple in a run of the DP algorithm. After evaluating the case *T *in the computation of *W*(*F*), one compares the maximum of the case to *W*(*F*). For example, a fragment (*i*, *j*; *k*, *l*) is 12G21-OD iff *W*(*i*, *j*; *k*, *l*) = max_*j'*, *k' *_*W *(*i*, *j' - *1; *k' *+ 1, *l*) + *W*(*j'*, *j*; *k*, *k'*).

In the following we show that for the maximization in a recursion case *T*, we do not need to consider *T'*-OD fragments as second fragment of the split, where *T' *is from a *T*-specific set of split types. As an example consider the recursion case 12G21, which splits fragments (*i*, *j*; *k*, *l*) into *F*_1 _= (*i*, *j' - *1; *k' *+1, *l*) and *F*_2 _= (*j'*, *j*; *k*, *k'*). Assume that *F*_2 _is 12G21-OD. Then we can show that every evaluation of *W*(*F*) where *W*(*F*) = *W*(*F*_1_) + *W *(*F*_2_) can be replaced by another at least equally good evaluation that splits *F *into ${F^{'}}_{1}$ and ${F^{'}}_{2}\subset F_{2}$, where ${F^{'}}_{2}$ is the second fragment in the 12G21-split of *F*_2_. However, note that the argument is split type specific and cannot be applied e.g. when *F*_2 _is 12G12-OD.

For sparsifying R&E, we define the following sets of split types.

$$\begin{array}{c} T_{12}^{\text{RE}}=\{12\}\\ T_{1212}^{\text{RE}}=\{12\text{G}2,\;\;12\text{G1,}\;\;1\text{G}21\}\\ T_{12\text{G}1}^{\text{RE}}=T_{1\text{G12}}^{\text{RE}}=T_{1\text{G2}1}^{\text{RE}}=\{12\}\\ T_{12\text{G2}}^{\text{RE}}=\{12\text{G}2\}\\ T_{12\text{G}21}^{\text{RE}}=\{12\text{G}2,\;\;1\text{G12,}\;\;12\text{G}21\}\\ T_{12\text{G}12}^{\text{RE}}=\{12\text{G}2,\;\;1\text{G21,}\;\;12\text{G}12\}\\ T_{1\text{G2}12}^{\text{RE}}=\{12\text{G1},\;\;1\text{G21,}\;\;12\text{G}21\}\\ T_{121\text{G}2}^{\text{RE}}=\{12\text{G}2,\;\;12\text{G1,}\;\;121\text{G}2\} \end{array}$$

These sets are defined such that in a recursion case *T*, whenever the second fragment of a split (*F*_1_, *F*_2_) of *F *can be optimally decomposed by a split of a type in $T_{T}^{\text{RE}}$, a different split $\left({F^{'}}_{1},{F^{'}}_{2}\right)$ of type *T *can be applied to *F*, where ${F^{'}}_{2}\subset F_{2}$. As we show later, this split will be just as good as (*F*_1_, *F*_2_) for computing *W*(*F*).

Then, one systematically obtains sparsified recursion equations *W'*(*i*, *j*) and *W'*(*i*, *j*; *k*, *l*) from the equations for *W*(*i*, *j*) and *W*(*i*, *j*; *k*, *l*) by replacing symbol *W *by *W' *and modifying them in the following way. For each case *T *in the recursion of *W*(*i*, *j*) and *W*(*i*, *j*; *k*, *l*) that maximizes over *W*(*F*_1_)+*W *(*F*_2_) for respective splits of the fragment *F *= (*i, j*) or *F *= (*i*, *j*; *k*, *l*), maximize only over fragments *F*_2 _that are not $T_{T}^{\text{RE}}$-OD. In an algorithm that evaluates the sparsified recursion, such non-$T_{T}^{\text{RE}}$-OD fragments correspond to entries of candidate lists. For example, case 12G21 of *W *is modified in the equation for *W' *(*i, j*, *k, l*) to

$$\underset{\begin{array}{l} j^{'},k^{'},(j^{'},j;k,k^{'})\\ not\;\;T_{12G21}^{RE}-OD \end{array}}{\max}\left(\begin{array}{l} W^{'}(i,j^{'}-1;k^{'}+1,l)\\ +W^{'}(j^{'},j;k,k^{'}) \end{array}\right)\;(12G21\;\;of\;W').$$

#### Theorem 1

*Let W be the matrix of the R&E recursion and W' its sparsified variant, then W*(1, |*S*|) = *W'*(1, *|S*|).

Proof We show for all 1 ≤ *i*, *j*, *k*, *l *≤ *|S|*, *W*(*i*, *j*) = *W'*(*i*, *j*) and *W*(*i*, *j*, *k*, *l*) = *W'*(*i*, *j*; *k*, *l*). First note that it holds that *W*(*i*, *j*) ≥ *W'*(*i*, *j*) and *W*(*i*, *j*; *k*, *l*) ≥ *W'*(*i*, *j*; *k*, *l*). The claim is shown by induction on the fragment size and a case distinction over recursion cases. For the case of split type 12, we show that

$$\begin{array}{l} \underset{j^{'}}{\max}W(i,j^{'}-1)+W(j^{'},j)=\\ \underset{j^{'},\;(j^{'},j)\;\;\text{not}\;\;T_{12}^{\text{RE}}-\;\text{OD}}{\max}W^{'}(i,j^{'}-1)+W^{'}(j^{'},j). \end{array}$$

Let (*j'*, *j*) be 12-OD for some *j' *: *i *≤ *j' *≤ *j*. By IH, it suffices to find a (smaller) fragment (*j''*, *j*), where *j'' > j *and *W*(*i*, *j'' - *1) + *W*(*j''*, *j*) ≥ *W*(*i*, *j' - *1) + *W*(*j'*, *j*). Either (*j'*, *j*) is not 12-OD or there is a *j''*, such that *W*(*j'*, *j*) = *W*(*j'*, *j'' - *1) + *W*(*j''*, *j*) and thus *W*(*i*, *j'' - *1)+*W*(*j''*, *j*) ≥ *W*(*i*, *j' - *1)+*W*(*j'*, *j*) because

$$\begin{array}{l} \;\;\;W(i,j^{\prime \prime }-1)+W(j^{\prime \prime },j)\\ \;\geq_{\Delta \text{-ineq}}\;W(i,j^{'}-1)+W(j^{'},j^{\prime \prime }-1)+W(j^{\prime \prime },j)\\ {=}_{12-\text{OD}}W(i,j^{'}-1)+W(j^{'},j). \end{array}$$

The triangle inequality (Δ-ineq) is an immediate consequence of the correctness of the recursion for *W*. Thus, for the decompositions of all recursion cases there holds such a corresponding inequation. Analogous arguments can be given for all other modified recursion cases. Exemplarily, we elaborate the argument for the complex case 12G21. Let *F*_1 _= (*i*, *j' - *1; *k' *+ 1, *l*) and *F*_2 _= (*j'*, *j*; *k*, *k'*), such that (*F*_1_, *F*_2_) is a split of type 12G21 of (*j*, *j*; *k*, *k*). We need to show for all $T_{12\text{G}21}^{\text{RE}}$-OD fragments *F*_2 _there are non-empty ungapped or 1-gap fragments ${F^{'}}_{1}$ and ${F^{'}}_{2}$, where F'1∪F'2=F2,F'1∩F'2=∅, and $W(F_{1}\cup {F^{'}}_{1})+W({F^{'}}_{2})\geq W(F_{1})+W(F_{2})$ and the split $\left(F_{1}\cup {F^{'}}_{1}\;,{F^{'}}_{2}\right)$ occurs in a recursion case of R&E. Again, either *F*_2 _is not $T_{12\text{G}21}^{\text{RE}}$-OD or one of the following cases applies. Case 1 (12G2): for some *j''*, *W*(*j'*, *j*; *k*, *k'*) = *W*(*j'*, *j'' - *1)+*W*(*j''*, *j*; *k*, *k'*). Then, the claim holds for ${F^{'}}_{1}=(j^{'},j^{\prime \prime }-1)$ and ${F^{'}}_{2}=(j^{\prime \prime },j;k,k^{'})$ by triangle inequality and split $\left(F_{1}\cup {F^{'}}_{1}\;,{F^{'}}_{2}\right)$ occurs in recursion case 12G21. Case 2 (2G21): for some *k''*, *W*(*j'*, *j*; *k*, *k'*) = *W*(*j'*, *j*; *k*, *k''*) + *W*(*k'' *+ 1, *k'*). The claim holds for ${F^{'}}_{2}=(j^{'},j;k,k^{\prime \prime })$. Case 3 (12G21): for some *j''*, *k''*, *W*(*j'*, *j*; *k*, *k'*) = *W*(*j'*, *j'' - *1; *k'' *+ 1, *k'*)+*W*(*j''*, *j*; *k*, *k''*). Again, this satisfies the claim by triangle inequality.

#### Algorithm

**Figure 3:**
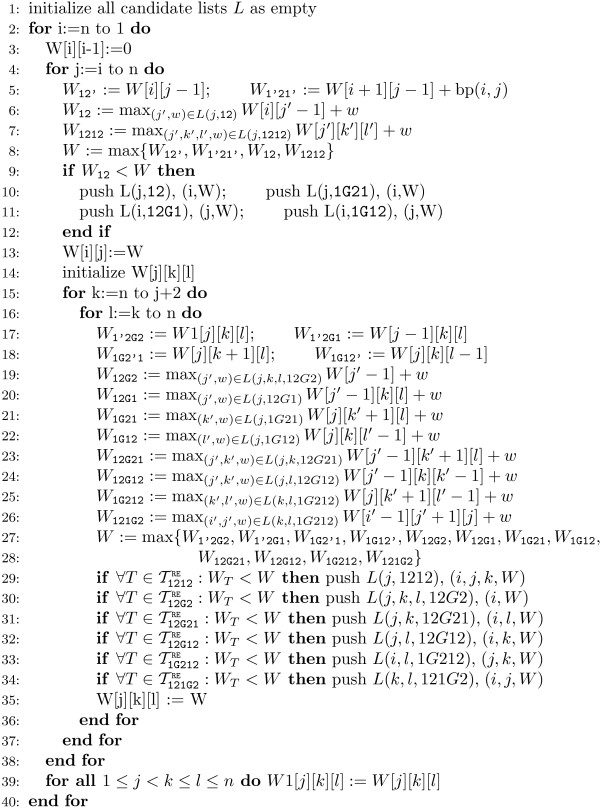
**Pseudocode for R&E-style base pair maximization**.

The recursion equation *W' *tailors a sparsified dynamic programming algorithm for the evaluation of *W' *(1, *|S|*) with very limited overhead. We maintain separate candidate lists for each sparsified recursion case. As already mentioned, the *T*-OD properties of each fragment *F *can be easily checked after evaluation of each case of *W*(*F*). A fragment is added to a candidate list for recursion case *T *iff it is not $T_{T}^{\text{RE}}$-OD. The maximizations are restricted to run only over the candidates in the respective candidate list. Their intended use dictates the exact nature of such candidate lists. For a case *T*, which splits a fragments *T *into *T*_1 _and *T*_2_, there are candidate lists for all boundaries of a fragment *T*_2 _that are not adjacent to boundaries of *T*_1 _due to split type *T*. The list entries are tuples of the adjacent boundaries and the fragment score for *T*_2_. In order to profit from a reduced number of candidates in space, we maintain two three-dimensional slices of the matrix for *W*(*i*, *j*; *k*, *l*), storing entries only for the current *i *and *i *+ 1. Scores *W*(*i*, *j*; *k*, *l*) for larger *i *are stored for candidates only. Pseudocode of the sparsified algorithm is given in Figure 3.

#### R&E Free Energy Minimization

Sparsification is analogously applied to the energy minimizing R&E algorithm. This algorithm distinguishes several additional matrices that contain minimal energies for fragments (*i*, *j*) or (*i*, *j*; *k*, *l*) under the condition that respectively the base pair (*i*, *j*) or base pairs (*i*, *l*) and (*j*, *k*) or one of them exist. Almost all decompositions in the recursion for these matrices are of discussed split types and are sparsified analogously. The only notable exception is due to internal loops. Internal loops require minimizing over all possible positions of the inner loop base pair, where commonly the loop size is restricted by a constant *K *such that minimizing takes constant time. However, handling inner loops requires access to entries of non-candidate fragments (*i'*, *j'*; *k'*, *l'*) for *i *≤ * i' *≤ *i *+ *K *+ 2. This is handled by maintaining matrix slices for *i *to *i *+ *K *+ 2 in *O*(*n*^3^) space, which preserves total space complexity.

#### Complexity Analysis

The described algorithm profits from sparsification in time and space. Compared to *O*(*n*^6^) time and *O*(*n*^4^) space of the unsparsified algorithm (for *n *= |*S*|), we obtain complexities in the number of candidates. Let *Z_T _*denote the maximal length of a candidate lists for case *T *and *Z *denote the total number of entries in all lists. Then, the time complexity is *O*(*n*^2^(*Z*_12 _+ *Z*_1212_) + *n*^4^(*Z*_12G2 _+ *Z*_12G1_+*Z*_1G21_+*Z*_1G12_+*Z*_12G21_+*Z*_12G12_+*Z*_1G212_+*Z*_121G2_)) and space complexity is *O*(*n*^3^+*Z*). In the worst case, *Z*_12_, *Z*_12G2_, *Z*_12G1_, *Z*_1G21 _and *Z*_1G12 _are *O*(*n*), *Z*_12G21_, *Z*_12G12_, *Z*_1G212_, *Z*_121G2 _are *O*(*n*^2^), and *Z*_1212 _is *O*(*n*^3^), finally *Z *is *O*(*n*^4^) in the worst case.

### Sparsification of the Dirks and Pierce Algorithm

Dirks and Pierce [12] present a pseudoknot prediction algorithm that takes *O*(*n*^5^) time and *O*(*n*^4^) space. Note that whereas Dirks and Pierce present their decomposition for computing the partition function, we sparsify the corresponding minimum free energy prediction algorithm. As mentioned in [15] this algorithm can be considered as a restriction of the algorithm by Rivas and Eddy to the cases

$$\begin{array}{cccccc} 12' & 1'2\text{G}2 & 12'\text{G}1 & 1\text{G}2'1 & 1\text{G}12' & \text{and}\\ 12 & 1212 & 12\text{G}2 & 12\text{G}1 & 1\text{G}21 & 1\text{G}12 \end{array}$$

with an additional case 1'2G21' that composes a gapped fragment (*i*, *j*; *k*, *l*) from a single base pair (*i*, *l*) and (*i *+ 1, *j*; *k*, *l - *1).

The non-constant cases 12, 1212, 12G2, 12G1, 1G21, and 1G12 can be sparsified exactly as the corresponding cases of the Rivas and Eddy algorithm with the following sets of split types:

$\begin{array}{cc} T_{12}^{DP}=\{12\} & T_{1212}^{DP}=\{12G2,12G1,1G21\}\\ T_{12G2}^{DP}=\{12G2\} & T_{12G1}^{DP}=T_{1G21}^{DP}=T_{1G12}^{DP}=\{12\} \end{array}$

Note that the additional case 1'2G21' does not need to be sparsified, because it is computed in constant time. Analogously to our discussion of the R&E algorithm, one obtains space and time complexities of the sparsified algorithm in terms of the length of candidate lists and the total number of candidates.

### Sparsification of the Akutsu and Uemura Algorithm

In this section we consider the pseudoknot prediction algorithm that was developed by Uemura *et al*. [9] based on tree adjoining grammars and later reformulated by Akutsu *et al*. [10] as dynamic programming algorithm. The algorithm predicts simple pseudo-knots in *O*(*n*^4^) time and *O*(*n*^3^) space. It can also be considered as a restriction of the algorithm by Rivas and Eddy. It is restricted to splits of the following types (again following the typing scheme of [15]):

$$\begin{array}{cccc} 12 & 121 & 12'\text{G2'}1 & 1\text{G}2'12'\\ 12'\text{G}1 & 1\text{G}2'1 & 1\text{G1}2' & 12'\text{G}2' \end{array}$$

and ommitted trivial, constant cases. Compared to the R&E algorithm, all cases that dominate the complexity are restricted to have only one possible split per instance (as indicated by the ' symbols; confer the additional case/split type of the algorithm by Dirks and Pierce). All non-constant cases, i.e. the first two rules, can still be sparsified analogous to sparsification of the algorithm of Rivas and Eddy using split type sets

$$T_{12}^{AU}=\{12\}\;and\;T_{121}^{AU}=\{12,121\}.$$

The restriction introduced by Akutsu and Uemura could be considered as a very simple, static form of sparsification. For each fragment annotated with symbol ', only one candidate (namely the smallest possible one) is considered. In contrast to sparsification as it is discussed in this paper, Akutsu's and Uemura's modification of the R&E algorithm reduces the worst-case complexity at the price of restricting the class of pseudoknots.

## Results and Discussion

In order to evaluate the effect of sparsification on pseudoknotted RNA secondary structure prediction, we implemented original and sparsified variants of the Reeder and Giegerich (R&G) algorithm.

### Data Set

We obtained all RNA sequences from Pseu-doBase [20], which are known to have some pseudo-knots in their secondary structures. This set contains 294 sequences that their length is distributed between 76 nt and 93399 nt. We randomly divided all long sequences into subsequences shorter than 1000 nt. Therefore the data set that we used in our experiments contains 1563 sequences with length between 76 nt and 1000 nt.

### Performance

**Figure 4:**
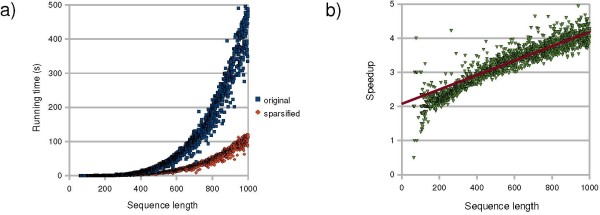
**Running times of the original and sparsified variants of the R&G algorithm**.

We applied both variants of the R&G algorithm to our data set. Figure 4 shows the running time of the algorithms on a server with Intel Core Duo CPU at 2.53 GHz and 4 GB RAM. The results in Figure 4 show that sparsification significantly improves the running time of the R&G algorithm. As the RNA sequences get longer, the relative performance of the sparsified algorithm (with respect to the non-sparsified ones) improves. Figure 4(b) shows the speedup of the sparsified algorithm, which fits well to a linear regression (*R*^2 ^= 0.84).

### Number of candidates

**Figure 5:**
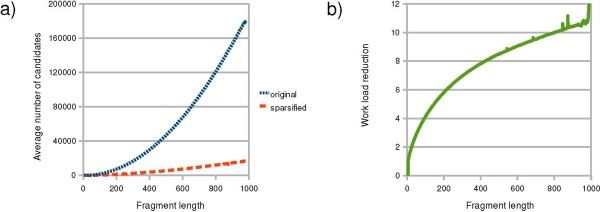
**Average number of (*i'*, *j'*) candidates in the original and sparsified variants of the R&G algorithm**.

For a better understanding of the effect of sparsification on the R&G algorithm, we measured the number of (*i'*, *j'*) pairs which are checked in each fragment [*i*, *j*] in both original and sparsified variants of the algorithm. Note that the number of (*i'*, *j'*) pairs is in order of *O*((*j - i*)^2^) in the worst case. Figure 5 shows the average number of (*i'*, *j'*) pairs on fragments of equal length which are checked by the two variants of the algorithm. As expected, this amount is significantly smaller for the sparsified algorithm compared to the original one. Moreover, we observe that as the fragments get longer, the difference between the average number of (*i'*, *j'*) pairs in the sparsified and the original algorithm increases. We define the work load per each fragment [*i*, *j*] as the number of candidate (*i'*, *j'*) pairs. Figure 5(b), shows a significant reduction of the work load in the sparsified algorithms. As it can be seen for subsequences of length 1000 nt, the work load by the sparsified algorithm is reduced by a factor of about 10 compared to the original algorithm. Note that the work load reduction at fragment length 1000 nt does not yield the same speedup for sequences of length 1000 nt (here this speedup is about 3.5, confer Figure 4(b)), because for a sequence of length *n*, all fragments of smaller length are processed by the algorithm.

## Conclusions

The presented work gives four examples for sparsification in the context of gap fragments and a complex recursion structure. We successfully sparsified the fastest and the most complex pseudo-knot structure prediction algorithm for RNA, as well as two algorithms with intermediate complexity. Since sparsification is similar in all these algorithms, the paper motivates further generalization of sparsification for systematic application to complex DP-algorithms as RNA structure prediction algorithms. Even more, by providing detailed examples the paper directly suggests such generalization. Our results from an implementation of the sparsified Reeder and Giegerich algorithm show a significant, presumably even linear, expected work load reduction due to sparsification. As future work, it would be interesting to develop optimizations for the partition function based variants of pseudoknot prediction where sparsification is not directly applicable.

## Competing interests

The authors declare that they have no competing interests.

## Authors' contributions

All authors developed the ideas for this project. MM, RS, and SW elaborated the technical contribution and wrote the paper. RS did the implementation and evaluation. All authors read and approved the final manuscript.
